# Videos With the Hashtag #vaping on TikTok and Implications for Informed Decision-making by Adolescents: Descriptive Study

**DOI:** 10.2196/30681

**Published:** 2021-10-25

**Authors:** Corey H Basch, Joseph Fera, Alessia Pellicane, Charles E Basch

**Affiliations:** 1 William Paterson University Wayne, NJ United States; 2 Lehman College Bronx, NY United States; 3 Teachers College Columbia University New York, NY United States

**Keywords:** vaping, TikTok, social media, misinformation, decision-making, adolescents, young adults, e-cigarettes, public health, informed decision-making

## Abstract

**Background:**

Despite the public health importance of vaping and the widespread use of TikTok by adolescents and young adults, research is lacking on the nature and scope of vaping content on this networking service.

**Objective:**

The purpose of this study is to describe the content of TikTok videos related to vaping.

**Methods:**

By searching the hashtag #vaping in the discover feature, ~478.4 million views were seen during the time of data collection. The first 100 relevant videos under that hashtag were used in this study. Relevance was determined by simply noting if the video was related in any way to vaping. Coding consisted of several categories directly related to vaping and additional categories, including the number of likes, comments, and views, and if the video involved music, humor, or dance.

**Results:**

The 100 videos included in the sample garnered 156,331,347 views; 20,335,800 likes; and 296,460 comments. The majority of the videos (n=59) used music and over one-third (n=37) used humor. The only content category observed in the majority of the videos sampled was the promotion of vaping, which was included in 57 videos that garnered over 74 million views (47.5% of cumulative views). A total of 42% (n=42) of the 100 videos sampled featured someone vaping or in the presence of vape pens, and these videos garnered over 22% (>35 million) of the total views.

**Conclusions:**

It is necessary for public health agencies to improve understanding of the nature and content of videos that attract viewers’ attention and harness the strength of this communication channel to promote informed decision-making about vaping.

## Introduction

Use of e-cigarettes or “vaping” functions by producing an aerosol when liquid nicotine is heated [1]. Liquid nicotine contains chemicals (eg, heavy metals such as nickel, tin, and lead; volatile organic compounds like benzene; the carcinogens acetaldehyde and formaldehyde; cadmium, a toxic metal; and ultrafine particles that can be inhaled deeply) and flavorings (eg, diacetyl, a chemical linked to the condition bronchiolitis obliterans, and diketone, also known to cause lung damage), which are inhaled into the lungs [2]. “E-cigarettes are not safe for youth, young adults, pregnant adults, as well as adults who do not currently use tobacco products, according the United States Centers for Disease Control and Prevention (CDC)” [1]. Additionally, “while e-cigarettes may have the potential to benefit some people and harm others, scientists still have a lot to learn about whether e-cigarettes are effective in helping adults quit smoking” [1]. Evidence suggests that vaping has negative health effects [3]. Current (2020) estimates indicate that 19.6% of high school students and 4.7% of middle school students in the United States reported present use of e-cigarettes [4]. A survey of adolescents in the United States revealed a positive association between frequency of social media use and exposure to e-cigarette messages across four different social media platforms [5]. Further, in a recent study of adolescents aged 13-18 years, an association was found between increased daily social media use and intent to use e-cigarettes, and that those who used social media more daily had a more positive outlook about e-cigarettes and sensed that e-cigarettes were less dangerous [6]. There have been studies of vaping on several social media websites. Researchers on Instagram found that e-cigarettes were promoted among youth [7] and that provaping content is prevalent [8]. Similar sentiment was noted on YouTube [9,10], with researchers noting the presence of beneficial health claims [11] and minimal Food and Drug Administration warnings [12]. In concert, studies of vaping content on Twitter determined that there was a high level of endorsement of vaping [13], and these were dominant forces [14].

TikTok, a social media platform, has had an exponential increase in popularity, with roughly 100 million monthly users in the United States and 689 million monthly users worldwide [15]. This platform allows for the uploading of short video segments, which often tend to be entertainment based. In the United States, the age groups that most commonly use TikTok are those aged 10-19 years (32.5%), followed by those 20-29 years of age (29.5%) [16]. Despite the public health importance of vaping and the widespread use of TikTok by adolescents and young adults, at the time this study was conducted (March 2021), we did not identify any published studies on the nature or scope of vaping on TikTok, thus identifying a gap in the literature. The purpose of this study is, therefore, to describe the content of posts on TikTok related to vaping.

## Methods

In March 2021, a cross-sectional, descriptive study was conducted. By searching the hashtag #vaping in the discover feature, ~478.4 million views were seen during the time of data collection. The first 100 relevant videos under that hashtag were used in this study. The coding sheet was based on a prior study of e-cigarettes conducted on a different social media platform [9], and the methods mirrored those of another TikTok study with a different focus [17]. Relevance was determined by simply noting if the video was related in any way to vaping. The coding categories included showing someone vaping or in the presence of vape pens, mentioned danger, mentioned/suggested long-term health effects, mentioned specific products, demonstrated how to make homemade vaping products, showed vape stores and/or purchasing vape products, showed vaping tricks (blowing smoke rings), contained information from medical professionals, mentioned safety, and contained misinformation. Additional categories included if the video involved music, humor, or dance. In addition to the number of videos associated with each category, the number of likes and comments were also documented. One individual (author AP) coded all videos, while a second individual (author CHB) coded a 10% random sample. Out of 380 total data points, the two reviewers differed in only 3, demonstrating high interrater reliability (κ=0.98). Descriptive statistics were calculated using Excel (Microsoft Corporation). Human participants were not included in this research, which was not reviewed by the Institutional Review Board (IRB) at William Paterson University; the study was deemed exempt by the IRB at Teachers College, Columbia University.

## Results

The 100 videos included in the sample garnered 156,331,347 views; 20,335,800 likes; and 296,460 comments (Table 1). The majority of the videos (n=59) used music and over one-third (n=37) used humor. The only content categories observed in the majority of the sample was “promoted vaping,” which was included in 57 videos that garnered over 74 million views (47.5% of cumulative views). Independent 1-tailed *t* tests (=.05) confirmed that using music or promoting vaping alone did not have a statistically significant association with whether a video was viewed, liked, or commented on. Even though the videos covering “mentioned danger” and “mentioned long-term health effects” were only covered in 38 and 30 videos, respectively, videos covering each of these categories garnered ~54% of the cumulative views (over 84 million). Although 42 of the videos featured someone vaping or in the presence of vape pens, these videos only garnered 22.67% (n=35,447,500) of the total views.

The following remaining characteristics were present in fewer than half but still over one-quarter (>25%) of the videos sampled: showing someone vaping or vape pens (n=42), mentioned dangers (n=38), used humor (n=37), and mentioned long-term effects (n=30). In these cases, too, independent 1-tailed *t* tests (α=.05) were performed to determine if the presence of this content was statistically associated with views, likes, or comments received. Only one test returned significant results (*P*<.05). Showing someone vaping or vape pens returned a statistically significant result (*P*=.02) with respect to video views.

**Table 1:** Observed content, views, likes, and comments of 100 TikTok videos related to vaping.

			Videos (N=100), n		Views (N=156,331,347), n (%)		Likes (N=20,335,800), n (%)		Comments (N=296,460), n (%)
Used music			59		69,398,247 (44.39)		7,450,600 (36.64)		72,004 (24.29)
Used humor			37		75,969,247 (48.60)		12,129,900 (59.65)		195,854 (66.06)
Used dance			2		3,700,000 (2.37)		601,800 (2.96)		8663 (2.92)
**Provaping content**									
	Promoted vaping	57		74,256,900 (47.50)		7,410,900 (36.44)		75,397 (25.43)	
	Showed someone vaping or in the presence of vape pens	42		35,447,500 (22.67)		4,182,600 (20.57)		39,682 (13.39)	
	Mentioned specific products	18		27,208,600 (17.40)		2,313,900 (11.38)		13,399 (4.52)	
	Demonstrated how to make homemade vaping products	15		25,059,200 (16.03)		1,327,700 (6.53)		3983 (1.34)	
	Contained misinformation	6		17,984,900 (11.50)		1,621,300 (7.97)		32,753 (11.05)	
**Antivaping content**									
	Mentioned dangers	38		84,911,247 (54.31)		12,684,700 (62.38)		228,753 (77.16)	
	Mentioned long-term health effects	30		84,316,147 (53.93)		12,208,700 (60.04)		219,399 (74.01)	
	Contained information from medical professionals	11		48,035,700 (30.73)		9,147,500 (44.98)		166,336 (56.11)	
	Mentioned safety	9		15,903,800 (10.17)		1,963,000 (9.65)		14,669 (4.95)	

## Discussion

This study demonstrates that the portrayal of vaping content is prevalent on TikTok. This is exemplified by the fact that 42 of the 100 videos in our sample showed someone vaping or in the presence of vape pens, and these videos garnered over 35 million views. Additionally troubling was the fact that more than half of the videos in the sample, which garnered over 74 million views, “promoted vaping.” On a positive note, 38 of the 100 videos mentioned the dangers of vaping, and 30 of the videos mentioned long-term health consequences; videos covering these topics attracted over 84 million views, the highest proportion of cumulative views of any coding category. Although there were 6 videos containing misinformation, there were 11 containing information from medical professionals.

Although the conclusions that can be drawn from this study are limited by the cross-sectional design, small and selective sample, and limited scope of information coded, the data show that a variety of information about vaping is being communicated and widely viewed on TikTok. This is particularly important since the majority of TikTok users are within an age range that makes them susceptible to both the influence of social media and experimentation with vaping. It is important to note that user agreements prohibit content that depicts use of alcohol, tobacco, or drugs by a minor [18]. However, the age of the person featured in each video was not estimated to avoid introducing the potential for error. This study fills a research gap by investigating a public health issue on an emerging video-sharing networking service. The necessity to learn more about coverage of vaping content on this platform is confirmed by the age of users and the popularity of the site. Public health agencies not only should be aware of and address provaping communications on TikTok and other social media but also should find ways to communicate effectively and help adolescents and young adults make informed decisions about vaping based on accurate and up-to-date scientific understanding. The widespread reach of videos addressing the dangers and long-term health effects of vaping suggests that TikTok users are interested in this content.

Social media may be viewed as a source of entertainment for users, and this is clearly one of its benefits. At the same time, TikTok and other social media have become a dominant communication channel through which people learn about health, form health-related beliefs, and connect with others who may reinforce health-compromising behaviors. It is, therefore, necessary for public health agencies to improve understanding of the nature and content of videos that attract viewers’ attention and to harness the strength of the platform to promote informed decision-making about vaping.

## Abbreviations

IRB: Institutional Review Board

## References

[1] (2021). About electronic cigarettes (e-cigarettes). Centers for Disease Control and Prevention.

[2] (2020). What's in an e-cigarette?. American Lung Association.

[3] Werner AK, Koumans EH, Chatham-Stephens K, Salvatore PP, Armatas C, Byers P, Clark CR, Ghinai I, Holzbauer SM, Navarette KA, Danielson ML, Ellington S, Moritz ED, Petersen EE, Kiernan EA, Baldwin GT, Briss P, Jones CM, King BA, Krishnasamy V, Rose DA, Reagan-Steiner S, Lung Injury Response Mortality Working Group (2020). Hospitalizations and deaths associated with EVALI. N Engl J Med.

[4] Wang TW, Neff LJ, Park-Lee E, Ren C, Cullen KA, King BA (2020). E-cigarette use among middle and high school students - United States, 2020. MMWR Morb Mortal Wkly Rep.

[5] Cho H, Li W, Shen L, Cannon J (2019). Mechanisms of social media effects on attitudes toward e-cigarette use: motivations, mediators, and moderators in a national survey of adolescents. J Med Internet Res.

[6] Vogel EA, Ramo DE, Rubinstein ML, Delucchi KL, Darrow S, Costello C, Prochaska JJ (2021). Effects of social media on adolescents' willingness and intention to use e-cigarettes: an experimental investigation. Nicotine Tob Res.

[7] Ketonen V, Malik A (2020). Characterizing vaping posts on Instagram by using unsupervised machine learning. Int J Med Inform.

[8] Alpert JM, Chen H, Riddell H, Chung YJ, Mu YA (2021). Vaping and Instagram: a content analysis of e-cigarette posts using the Content Appealing to Youth (CAY) Index. Subst Use Misuse.

[9] Basch CH, Mongiovi J, Hillyer GC, MacDonald Z, Basch CE (2016). YouTube videos related to e-cigarette safety and related health risks: implications for preventing and emerging epidemic. Public Health.

[10] Gao Y, Xie Z, Sun L, Xu C, Li D (2020). Electronic cigarette-related contents on Instagram: observational study and exploratory analysis. JMIR Public Health Surveill.

[11] Paek H, Kim S, Hove T, Huh JY (2014). Reduced harm or another gateway to smoking? source, message, and information characteristics of E-cigarette videos on YouTube. J Health Commun.

[12] Jones DM, Guy MC, Soule E, Sakuma KK, Pokhrel P, Orloff M, Trinidad D, Smith D, Browley S, Walker AP, Bullock S, Eissenberg T, Fagan P (2021). Characterization of electronic cigarette warning statements portrayed in YouTube videos. Nicotine Tob Res.

[13] McCausland K, Maycock B, Leaver T, Wolf K, Freeman B, Jancey J (2020). E-cigarette advocates on Twitter: content analysis of vaping-related tweets. JMIR Public Health Surveill.

[14] Cole-Lewis H, Pugatch J, Sanders A, Varghese A, Posada S, Yun C, Schwarz M, Augustson E (2015). Social listening: a content analysis of e-cigarette discussions on Twitter. J Med Internet Res.

[15] Iqbal M (2021). TikTok revenue and usage statistics (2021). Business of Apps.

[16] (2021). Distribution of TikTok users by age group. Statista.

[17] Basch CH, Meleo-Erwin Z, Fera J, Jaime C, Basch CE (2021). A global pandemic in the time of viral memes: COVID-19 vaccine misinformation and disinformation on TikTok. Hum Vaccin Immunother.

[18] (2020). Community guidelines. TikTok.

